# Comparison of the perinatal outcomes of expected high ovarian response patients and normal ovarian response patients undergoing frozen-thawed embryo transfer in natural/small amount of HMG induced ovulation cycles

**DOI:** 10.1186/s12889-024-17725-5

**Published:** 2024-01-22

**Authors:** Wenjuan Zhang, Zhaozhao Liu, Bijun Wang, Manman Liu, Jiaheng Li, Yichun Guan

**Affiliations:** Reproduction Center, The Third Affiliated Hospital of ZhengZhou University, Henan, China

**Keywords:** Expected high ovarian response, Normal ovarian response, Natural cycles, Maternal and neonatal outcomes

## Abstract

**Background:**

Due to the high risk of complications in fresh transfer cycles among expected high ovarian response patients, most choose frozen-thawed embryo transfer (FET). There are currently few researches on whether the FET outcomes of expected high ovarian response patients with regular menstrual cycles are similar to those of normal ovarian response. Therefore, our objective was to explore and compare pregnancy outcomes and maternal and neonatal outcomes of natural FET cycles between patients with expected high ovarian response and normal ovarian response with regular menstrual cycles based on the antral follicle count (AFC).

**Methods:**

This retrospective cohort study included 5082 women undergoing natural or small amount of HMG induced ovulation FET cycles at the Reproductive Center of the Third Affiliated Hospital of Zhengzhou University from January 1, 2017, to March 31, 2021. The population was divided into expected high ovarian response group and normal ovarian response group based on the AFC, and the differences in patient characteristics, clinical outcomes and perinatal outcomes between the two groups were compared.

**Results:**

Regarding clinical outcomes, compared with the normal ovarian response group, patients in the expected high ovarian response group had a higher clinical pregnancy rate (57.34% vs. 48.50%) and live birth rate (48.12% vs. 38.97%). There was no difference in the early miscarriage rate or twin pregnancy rate between the groups. Multivariate logistic regression analysis suggested that the clinical pregnancy rate (adjusted OR 1.190) and live birth rate (adjusted OR 1.171) of the expected high ovarian response group were higher than those of the normal ovarian response group. In terms of maternal and infant outcomes, the incidence of very preterm delivery in the normal ovarian response group was higher than that in the expected high ovarian response group (0.86% vs. 0.16%, adjusted OR 0.131), Other maternal and infant outcomes were not significantly different. After grouping by age (< 30 y, 30–34 y, 35–39 y), there was no difference in the incidence of very preterm delivery among the age subgroups.

**Conclusion:**

For patients with expected high ovarian response and regular menstrual cycles undergoing natural or small amount of HMG induced ovulation FET cycles, the clinical and perinatal outcomes are reassuring. For patients undergoing natural or small amount of HMG induced ovulation FET cycles, as age increases, perinatal care should be strengthened during pregnancy to reduce the incidence of very preterm delivery.

**Supplementary Information:**

The online version contains supplementary material available at 10.1186/s12889-024-17725-5.

## Introduction

There are individual differences in ovarian responsiveness during controlled ovarian hyperstimulation. Affected by exogenous gonadotropin (Gn) stimulation, the number and quality of follicles differ in different patients. According to the sensitivity of the ovaries to Gn during ovarian hyperstimulation, ovarian responsiveness can be divided into high ovarian response, normal ovarian response, and poor ovarian response. Patients with high ovarian response are prone to high serum oestradiol (E_2_) levels, excessive follicular development, and ovarian hyperstimulation syndrome (OHSS) [1] after Gn stimulation. In severe cases, the high ovarian response can even be life-threatening. It is essential to accurately identify individuals with high ovarian response and reduce the occurrence of adverse reactions during controlled ovarian hyperstimulation. At present, there is no unified standard for determining and predicting high ovarian response on a global scale, but there are still several indicators that are helpful in predicting the high ovarian response [2, 3]: ① age ≤ 35 y; ② antral follicle count(AFC) ≥ 15; and ③ anti-Müllerian hormone (AMH) level > 4.5 mg/L. This study used the commonly used prediction standard of an AFC ≥ 15 [4].

The Zeilmaker team reported the first successful live birth after frozen-thawed embryo transfer (FET) [5]. The number of FET cycles is steadily increasing globally [6, 7]. Recent studies had suggested that the success rate of FET cycles was not inferior to that of fresh embryo transfer cycles [8, 9], while two high-quality randomized controlled trials (RCTs) had suggested that FET cycles can achieve a higher live birth rate (LBR) than fresh transfer cycles [10, 11]. Moreover, some studies found that FET cycles had a higher pregnancy rate and a lower incidence of complications [12, 13], making FET a safe and effective measure for patients with high ovarian response.

The endometrial preparation protocol optimizes the success rate of FET by synchronizing endometrial receptivity with the embryonic development stage. There are three commonly used FET protocols: natural cycles, induced ovulation cycles, and hormone replacement therapy (HRT) cycles. An RCT study involving 1032 patients with regular menstrual cycles showed that there was no statistically significant difference in the LBR, clinical pregnancy rate (CPR) or cost between improved natural cycles and HRT cycles, but the rate of cycle cancellation was higher in HRT cycles [9]. However, because exogenous oestrogen and progestogen are used in HRT to prepare the endometrium and an endogenous corpus luteum is lacking, pregnant women receiving HRT have an increased risk of hypertensive disorders of pregnancy (HDP) and placenta implantation abnormalities [14, 15], and their newborns have an increased risk of low birth weight (LBW) and macrosomia [16]. Therefore, HRT cycles are not the first choice in clinical treatment for women with regular menstrual cycles. Another RCT that compared pregnancy outcomes between patients with regular menstrual cycles undergoing human menopausal gonadotrophin (HMG)-induced ovulation cycles and natural FET cycles showed that there was no significant difference in the embryo implantation rate or LBR between the two groups; therefore, for women with regular menstrual cycles, natural cycles with less intervention are the first choice [17]. For women with normal ovulation, natural cycles seem to be the optimal choice due to the following: changes in the endometrium that are most in line with physiological conditions, the lack of use of exogenous hormones, low costs, and relatively reassuring maternal and infant outcomes [14, 18].

Induced ovulation cycles are mainly used for patients with ovulation disorders, but for patients with follicular dysplasia during natural cycle monitoring, the use of a small amount of HMG to induce ovulation is a good choice [19]. In this study, natural FET cycles were preferred for women with regular menstrual cycles. However, for patients who did not have a dominant follicle (diameter of ≥ 10 mm) on the 10th day of menstruation or had follicular dysplasia, low-dose HMG was used to induce ovulation.

Due to the limited research on the FET outcomes of individuals with expected high ovarian response who have a regular menstrual cycle, the main purpose of this study was to compare the clinical and perinatal outcomes of natural FET cycles between women with expected high ovarian response and normal ovarian response to provide guidance for clinical practice.

## Materials and methods

### Study design and participants

Institutional review board approval for this retrospective cohort study was obtained from the Ethics Committee of the Third Affiliated Hospital of Zhengzhou University (reference: 2022-61). A total of 5082 women who underwent FET cycles from January 2017 to March 2021 in our department were enrolled. We included women with normal ovulation undergoing natural or small amount of HMG induced ovulation FET cycles after in vitro fertilization (IVF)/intracytoplasmic sperm injection (ICSI).

All of the enrolled patients met the following criteria: (1) aged < 40 y, (2) had a menstrual cycles lasting 30 ± 7 days, and (3) had an AFC >5. The exclusion criteria were (1) preimplantation genetic testing, (2) donor cycles, (3) missing data, (4) transfer cancellation due to various reasons, and (5) stillbirth.

### Endometrial preparation

For patients undergoing natural cycles, follicle monitoring began on day 10 of the menstrual cycle to evaluate the development of ovarian follicles. If the follicle diameter was < 10 mm or follicular development was poor, 37.5–75 IU daily HMG (Lizhu Pharmaceutical Trading Co., China) was added as appropriate for follicular development (the total dose of HMG was < 300 IU). When the diameter of the dominant follicle reached 14–20 mm, a serum sample was obtained to measure oestradiol (E_2_), progesterone (P), and luteinizing hormone (LH) levels. When the dominant follicle reached 14 mm, the serum LH level indicated that ovulation was about to occur, the E_2_ level was more than 150 pg/mL and the endometrial thickness was more than 7 mm, 10 000 IU hCG was injected (Lizhu Pharmaceutical Trading Co., China). All patients were required to sign written informed consent after receiving information about hMG.

After the hCG injection, routine corpus luteum support, namely, Oral dydrogesterone (2 times daily, 10 mg once) (Abbott Co. America) and 90 mg of a progesterone sustained-release vaginal gel (Merck Co. Germa), was given. Three or five days after endometrial development with corpus luteum support, embryo transfer was carried out by abdominal ultrasound. Corpus luteum support was performed at least until 55 days after transplantation if pregnancy occurred.

### Data collection and outcome definitions

Patient characteristics such as age, body mass index (BMI), type of infertility, indication for IVF, duration of infertility, basal Anti-Mullerian hormone(AMH) level, AFC, endometrial thickness, the number of transferred embryos, developmental stage of the embryos, whether pregnancy or live birth occurred, and singleton or twin pregnancy were obtained from the electronic case system of our department.

For the patients with a gestational sac echo and a single live birth after embryo transfer, pregnancy complications were recorded via telephone follow-up performed by the fixed nurse in our department, and the perinatal outcomes and neonatal outcomes were recorded and classified according to the information provided by the patients.

Early spontaneous abortion was defined as a clinical pregnancy that failed to reach the 12th gestational week. Live birth was defined as the birth of a live child after 28 weeks of gestation for each embryo transfer cycle. Very preterm delivery (VPTD), preterm delivery (PTD), term delivery, and post-term delivery were defined as delivery at < 32 weeks of gestation, delivery at < 37 weeks of gestation, delivery between 37 weeks and 41 weeks, and delivery at > 41 weeks of gestation, respectively. The birth weights of singleton live-born infants were classified as follows: low birth weight (LBW, < 2500 g), small for gestational age (SGA, < 10th percentile for gestational age) [20], macrosomia (≥ 4000 g), and large for gestational age (LGA, > 90th percentile for gestational age) [20].

### Statistical analysis

All the statistical management and analyses were performed using SPSS software, version 22.0. The one-sample Kolmogorov − Smirnov (K-S) test was used to check for normality. Continuous variables are expressed as the mean ± SD, and Student’s t test was used to assess between-group differences. Categorical variables are represented as the number (n) and percentage (%) of cases. Means from chi-square analyses were used to assess differences between the groups. Multiple logistic regression was applied to further analyse different items. Unadjusted odds ratios(ORs) and adjusted odds ratios(aORs) with 95% confidence intervals (CIs) were calculated. Statistical significance was set at *P* < 0.05.

## Results

There were 2107 patients with normal ovarian response in Group A(5 < AFC < 15) and 2975 patients with expected high ovarian response in Group B (AFC ≥ 15). When comparing the basic characteristics between the two groups, we found that Group B was younger, had a lower proportion of the fallopian tube factors, had a shorter duration of infertility, had a higher proportion of primary infertility and male factors, and had a higher basal AMH level than Group A, which was due to the higher AFC in Group B (Table 1).

**Table 1:** Patient clinical characteristics

Characteristics	Group A (5<AFC<15) 2107	Group B (AFC ≥ 15) 2975	*P* value
Female age (y)	32.55 ± 3.91	30.68 ± 3.80	0.000
BMI (kg/m2)	23.26 ± 2.96	23.33 ± 3.17	0.383
Type of infertility			0.000
Primary infertility	37.30%(786/2107)	42.22%(1256/2975)	
Secondary infertility	62.70%(1321/2107)	57.78%(1719/2975)	
Indication for IVF			
Tubal factor	63.08(1329/2107)	55.83%(1661/2975)	0.000
Ovulatory dysfunction	3.84%(81/2107)	4.84%(144/2975)	0.089
Endometriosis	0.43%(9/2107)	0.24%(7/2975)	0.229
Male factor	22.97%(484/2107)	29.11%(866/2975)	0.000
Others	10.72%(204/2107)	9.98%(297/2975)	0.723
Duration of infertility (y)	3.48 ± 2.98	3.07 ± 2.39	0.000
Number of previous FET failures	0.70 ± 0.87	0.65 ± 0.82	0.028
Number of previous spontaneous abortions	0.11 ± 0.32	0.12 ± 0.34	0.335
Basal AMH(pmol/L)	14.45 ± 10.86	31.17 ± 23.45	0.000

AMH: Anti-Mullerian hormone; BMI: Body mass index

In terms of the clinical and embryological characteristics, compared to Group A, in Group B, the rate of HMG-induced cycles was higher, the endometrium was thicker, and the rates of single embryo transfer and blastocyst transfer were relatively higher. The CPR and LBR in Group B were higher than those in Group A, while there were no significant differences in the early spontaneous abortion rate or twin pregnancy rate between the two groups (Tables 2 and 3).

**Table 2:** Patient clinical and embryological characteristics

Characteristics	Group A (5<AFC<15) 2107	Group B (AFC ≥ 15) 2975	*P* value
HMG-induced cycle rate	13.91%(293/2107)	17.68%(526/2975)	0.000
Endometrial thickness on the day of embryo transfer (mm)	9.60 ± 1.64	9.74 ± 1.67	0.004
Thin endometrium	1.80%(38/2107)	1.34%(40/2975)	0.190
Number of transferred embryos			0.000
One	51.28%(1080/2107)	59.56%(1772/2975)	
Two	48.74%(1027/2107)	40.44(1203/2975)	
Development stage of the embryo			
D3	43.85%(924/2107)	28.81%(857/2975)	0.000
D5/D6	54.11%(1140/2107)	68.84%(2048/2975)	0.000
Sequential transfer	2.04%(43/2107)	2.35%(70/2975)	0.457

**Table 3:** Clinical outcomes

	Group A (5<AFC<15) 2107	Group B (AFC ≥ 15) 2975	*P* value
Clinical pregnancy rate(CPR)	48.50%(1022/2107)	57.34%(1706/2975)	0.000
Early spontaneous abortion rate	15.58%(159/1022)	12.72%(217/1706)	0.037
Live birth rate(LBR)	38.97%(821/2107)	48.12%(1430/2975)	0.000
Singleton pregnancy	85.99%(706/821)	87.06%(1245/1430)	0.472
Twin pregnancy	14.01%(115/821)	12.94%(185/1430)	

Due to the differences in the CPR and LBR between the two groups, further logistic regression was performed and showed that the CPR and LBR in Group B were higher than those in Group A (Table 4 aOR 1.190, 95% CI 1.048–1.352; aOR 1.171, 95%CI 1.030–1.331).

**Table 4:** Logistic regression of the pregnancy outcomes between the two groups

	Adjusted OR (95% CI)	*P* value
Clinical pregnancy rate	1.190(1.048–1.352)	0.007
Early spontaneous abortion rate	1.045(0.823–1.327)	0.716
Live birth rate	1.171(1.030–1.331)	0.016

Note: The analysis was adjusted for female age, type of infertility, duration of infertility, the number of previous FET failures, BMI, whether HMG was added, endometrial thickness on the day of embryo transfer, the number of transferred embryos, and the developmental stage of the embryo

CI: Confidence interval

We separately analysed the patients with a gestational sac echo and a singleton live birth after embryo transfer. There were 694 patients in Group A and 1213 patients in Group B. We mainly analysed the maternal and neonatal outcomes. We found that there were no significant differences in perinatal outcomes, including the incidence of VPTD, PTD, post-term delivery, LBW, macrosomia, SGA, LGA, GDM, HDP, placenta previa, and congenital malformations, neonatal weight, or the newborn sex ratio between the two groups (Table 5).

**Table 5:** Perinatal and neonatal outcomes of singleton live births

	Group A (5<AFC<15) 694	Group B (AFC ≥ 15) 1213	*P* value
VPTD	0.86%(6/694)	0.16%(2/1213)	0.057
PTD	6.20%(43/694)	4.86%(59/1213)	0.214
Post-term delivery	0.29%(2/694)	0.25%(3/1213)	1.000
Neonatal weight (g)	3394.55 ± 495.39	3690.69 ± 9103.18	0.392
Newborn sex			0.159
Male	52.31%(363/694)	55.65%(675/1213)	
Female	47.69%(331/694)	44.35%(538/1213)	
LBW	3.46%(24/694)	2.72%(33/1213)	0.363
Macrosomia	11.38%(79/694)	11.95%(145/1213)	0.710
SGA	6.20%(43/694)	6.43%(78/1213)	0.840
LGA	17.58%(122/694)	16.41%(199/1213)	0.510
GDM	8.36%(58/694)	7.26%(88/1213)	0.384
HDP	3.17%(22/694)	3.38%(41/1213)	0.805
HDP + GDM	0.43%(3/694)	0.25%(3/1213)	0.788
Placenta previa	0.58%(4/694)	0.49%(6/1213)	1.000
Congenital malformation	1.30%(9/694)	0.66%(8/1213)	0.154

VPTD: very preterm delivery; PTD: preterm delivery; LBW: low birth weight

SGA: small for gestational age; LGA: large for gestational age

GDM: gestational diabetes mellitus; HDP: hypertensive disorders of pregnancy

PROM: premature rupture of membranes

Further logistic regression showed that the incidence of VPTD in Group B was lower than that in Group A. (Table 6 aOR 0.131, 95%CI 0.021–0.828). There were no differences in the other perinatal or neonatal outcomes.

**Table 6:** Logistic regression of the perinatal and neonatal outcomes between the two groups

	Adjusted OR (95% CI)	*P* value
VPTD	0.131(0.021–0.828)	0.031
PTD	0.810(0.516–1.272)	0.361
Post-term delivery	3.368(0.342–33.199)	0.298
LBW	0.718(0.395–1.305)	0.277
Macrosomia	0.942(0.681–1.303)	0.718
SGA	1.169(0.752–1.817)	0.488
LGA	0.912(0.689–1.207)	0.521
GDM	1.046(0.706–1.550)	0.823
HDP	1.052(0.573–1.931)	0.869
HDP + GDM	1.411(0.195–10.231)	0.733
Placenta previa	1.945(0.442–8.568)	0.379
Congenital malformation	0.466(0.156–1.393)	0.172

Due to the significant differences in age between the two groups, we further stratified by age (< 30 y, 30–34 y, 35–39 y). The results showed that in the subgroup aged 30-34y, when compared to the normal ovarian response patients, the CPR and LBR significantly increased among the expected high ovarian response patients, which was consistent with the overall trend. Moreover, there were no significant differences in the incidence of VPTD or PTD between expected high ovarian response patients and normal ovarian response patients among the different age subgroups (Table 7).

**Table 7:** Logistic regression of the pregnancy outcomes between expected high ovarian response patients and normal ovarian response patients among the different age subgroups

	Age<30y		30y ≤ Age ≤ 34y		35y ≤ Age ≤ 39y	
	Adjusted OR (95% CI)	*P* value	Adjusted OR (95% CI)	*P* value	Adjusted OR (95% CI)	*P* value
Clinical pregnancy rate	1.167(0.936–1.455)	0.169	1.307(1.097–1.557)	0.003	1.180(0.930–1.498)	0.173
Live birth rate	1.216(0.979–1.511)	0.078	1.254(1.052–1.495)	0.012	1.144(0.890–0.906)	0.294
VPTD	0.092(0.008–1.029)	0.053	0.282(0.024–3.286)	0.313	0.000(0.000-4.189)	0.959
PTD	0.531(0.234–1.207)	0.131	1.067(0.565–2.018)	0.841	1.259(0.521–3044)	0.609

Note: The analysis was adjusted for female age, type of infertility, duration of infertility, the number of previous FET failures, BMI, whether HMG was added, endometrial thickness on the day of embryo transfer, the number of transferred embryos, and the developmental stage of the embryo

CI: Confidence interval VPTD: very preterm delivery; PTD: preterm delivery; LBW: low birth weight

VPTD and PTD are both perinatal outcomes of singleton live births

## Discussion

Our study showed that when compared to patients with normal ovarian response, those with expected high ovarian response with regular menstrual cycles who were undergoing natural FET cycles had an increased CPR and LBR. In terms of maternal and infant outcomes, the expected high ovarian response group showed a decreasing trend in the incidence of VPTD. When age was further stratified, there was no difference in the incidence of VPTD among the different age subgroups.

A retrospective study involving 5070 FET cycles revealed that for women with regular menstrual cycles, the CPR and LBR of women undergoing HMG-induced ovulation cycles were higher than those of women undergoing natural cycles [19]. A study by Peeraer et al. also suggested that, in women with normal ovulation undergoing FET cycles, the LBR in the low-dose HMG-induced ovulation group was higher than that in the natural-cycle group. In this study, the rate of HMG-induced cycles in the expected high ovarian response group was relatively high, which may be the reason for the increase in the CPR and LBR based on the literature. In principle, this may be due to the promotion of endogenous hormone secretion and endometrial growth, the increase in endometrial thickness and receptivity, and the facilitation of implantation caused by ovarian stimulation, thus increasing the CPR (21–22). In addition, an expected increase in endometrial thickness was observed in the high ovarian response group. Several studies have shown that the CPR increases with increasing endometrial thickness. Endometrial thickness may be an independent factor affecting pregnancy [23, 24]. The underlying mechanism may be that as the thickness of the endometrium increases, the resistance of uterine artery blood flow decreases, which in turn affects the remodelling of spiral artery blood vessels, increasing the blood supply to the placenta and leading to good pregnancy and perinatal outcomes [25]. This may also be another reason for the better clinical outcomes in the expected high ovarian response group.

A retrospective study in 2021 revealed that among women undergoing FET, the incidence of PTD and VPTD in women undergoing induced ovulation cycles was lower than that in women undergoing natural cycles and HRT cycles [14]. In this study, it was shown that the expected high ovarian response group had an increased rate of HMG-induced ovulation cycles, which may be a possible reason for the reduced risk of VPTD. It is speculated that high levels of steroid hormones during the stimulation cycle may induce abnormal endometriosis and placental abnormalities, thereby affecting the timing of delivery through epigenetic mechanisms [20], however the specific mechanism is still unclear. Of course, due to the small number of included women with VPTD, there may be a risk of bias.

As is well known, female age is an important predictor of assisted reproductive technology(ART) clinical outcomes. Many studies had shown that advanced age (over 40 years old) had a negative impact on pregnancy outcomes(26–27). As age increases, ovarian function decreases, oocyte quality decreases [28], and the embryo aneuploidy rate increases [29]. Additionally, abnormal endometrial function, and degeneration of multiple organ function can occur in women of advanced maternal age [30]. All the above-mentioned factors would affect the development of the embryos and cause adverse effects on the newborns, leading to a decrease in LBR and adverse perinatal outcomes. Many studies had revealed a negative correlation between maternal age and neonatal outcomes (31–32). A meta-analysis that included 10 studies suggested that older mothers (≥ 35 years old) had worse perinatal outcomes such as higher rates of PTD, LBW, higher rates of Neonatal Intensive Care Unit admission and worse Apgar scores. This may be due to a reduced cardiovascular reserve and an increased risk of preeclampsia and gestational diabetes in elderly women (33–34), which can lead to the poor placental perfusion [35], that is, increased age may be a risk factor for VPTD. This was also consistent with our research conclusion. The slightly higher VPTD rate in the normal ovarian response group may be related to the older age of that group.

Our research advantage lies in exploring the pregnancy outcomes of natural FET cycles between women with expected high ovarian response and those with normal ovarian response, as well as in comparing maternal and infant outcomes. The sample size was relatively large, which can provide certain guidance for clinical practice.

However, several limitations associated with this study warrant mention: (1) This was a retrospective study with some deviation; hence, additional prospective research is needed to verify our results. (2) Patients with diabetes and hypertension were not excluded, but blood pressure and blood glucose levels were controlled normally before FET was performed, which might have led to some inaccuracies in the results. (3) Because data on maternal complications and offspring outcomes were obtained by telephone and reported by patients, some data were incomplete or missing.

In summary, for patients with expected high ovarian response with regular menstrual cycles who are undergoing natural FET cycles, although the risk of follicular dysplasia requiring the addition of HMG is increased, the maternal and infant outcomes are reassuring. For patients undergoing natural or small amount of HMG induced ovulation FET cycles, as age increases, perinatal care should be strengthened during pregnancy to reduce the risk of very preterm delivery.

### Electronic supplementary material

Below is the link to the electronic supplementary material.

Supplementary Material 1

## Data Availability

The data sets used and/or analysed during the current study are available from the corresponding author upon reasonable request.
