# Supplementary material for: Comparison of the perinatal outcomes of expected high ovarian response patients and normal ovarian response patients undergoing frozen-thawed embryo transfer in natural/small amount of HMG induced ovulation cycles
Source: BMC Public Health. 2024 Jan 22;24:259. doi: 10.1186/s12889-024-17725-5 (PMC10804831; doi:10.1186/s12889-024-17725-5)
Supplement: Supplementary file 1 — Supplementary Material 1 [file 12889_2024_17725_MOESM1_ESM.docx]

**Table 8.** Patient clinical characteristics and clinical outcomes of expected high ovarian response patients and normal ovarian response patients among the different age subgroups

|  | Age＜30y | | | 30y≤Age≤34y | | | 35y≤Age≤39y | | |
| --- | --- | --- | --- | --- | --- | --- | --- | --- | --- |
|  | Group A1  (482) | Group B1  (1169) | *P* | Group A2  (912) | Group B2  (1297) | *P* | Group A3  （713） | Group B3  （509） | *P* |
| Female age (y) | 27.32±1.83 | 27.01±1.98 | 0.003 | 31.85±1.38 | 31.65±1.32 | 0.001 | 36.97±1.38 | 36.65±1.38 | 0.000 |
| BMI (kg/m2) | 22.75±3.11 | 23.10±3.19 | 0.040 | 23.03±2.81 | 23.26±3.13 | 0.077 | 23.89±2.93 | 24.05±3.16 | 0.344 |
| Duration of infertility (y) | 2.71±1.91 | 2.62±1.74 | 0.087 | 3.37±2.62 | 3.21±2.44 | 0.152 | 4.14±3.77 | 3.73±3.23 | 0.042 |
| Number of previous FET failures | 0.58±0.73 | 0.60±0.76 | 0.616 | 0.67±0.83 | 0.66±0.83 | 0.678 | 0.82±0.99 | 0.74±0.91 | 0.151 |
| Number of previous spontaneous abortions | 0.11±0.32 | 0.10±0.30 | 0.504 | 0.10±0.30 | 0.13±0.35 | 0.011 | 0.13±0.36 | 0.14±0.37 | 0.618 |
| Basal AMH（pmol/L） | 15.75±10.88 | 32.49±25.78 | 0.000 | 14.45±9.47 | 31.35±23.26 | 0.000 | 13.58±12.33 | 27.68±17.15 | 0.000 |
| Number of transferred embryos | 1.45±0.50 | 1.42±0.49 | 0.254 | 1.47±0.50 | 1.38±0.49 | 0.000 | 1.54±0.50 | 1.43±0.50 | 0.000 |
| HMG-induced cycle rate | 14.94%（72/482） | 20.79%（243/1169） | 0.0062 | 14.58%  (133/912) | 15.73%  (204/1297) | 0.461 | 12.34%  （88/713） | 15.52%  （79/509） | 0.111 |
| Endometrial thickness on the day of  embryo transfer (mm) | 9.89±1.66 | 9.91±1.67 | 0.848 | 9.62±1.62 | 9.69±1.70 | 0.331 | 9.40±1.62 | 9.51±1.58 | 0.235 |
| Clinical pregnancy rate | 56.64%（273/482） | 61.51%（719/1169） | 0.066 | 48.57%  (443/912) | 56.69%  (735/1297) | 0.000 | 42.92%  （306/713） | 49.51%  （252/509） | 0.023 |
| Live birth rate | 48.13%（232/482） | 53.89%（630/1169） | 0.033 | 40.35%  (368/912) | 47.19%  (612/1297) | 0.001 | 31.00%  （221/713） | 36.94%（188/509） | 0.030 |
| VPTD | 1.59%  (3/189) | 0.19%  (1/523) | 0.102 | 0.65%  (2/310) | 0.19%  (1/524) | 0.645 | 0.51%  （1/195） | 0  （0/166） | 0.267 |
| PTD | 5.82%  (11/189) | 3.25%  (17/523) | 0.119 | 5.48%  (17/310) | 5.73%  (30/524) | 0.884 | 7.69%（15/195） | 7.23%（12/166） | 0.868 |

Note: AMH: Anti-Mullerian hormone; BMI: Body mass index;

VPTD: very preterm delivery; PTD: preterm delivery

VPTD and PTD are both perinatal outcomes of singleton live births

Group A1,A2,A3 included normal ovarian response patients

Group B1,B2,B3 included expected high ovarian response patients
